# Health condition and job status interactions: econometric evidence of causality from a French longitudinal survey

**DOI:** 10.1186/s13561-019-0220-3

**Published:** 2019-01-31

**Authors:** Eric Delattre, Richard K. Moussa, Mareva Sabatier

**Affiliations:** 1 ThEMA, Université de Cergy-Pontoise, 33 bd du Port, Cergy-Pontoise, 95011 France; 2 Ecole Nationale Supérieure de Statistique et d’Economie Appliquée, Boulevard des Universités, Cocody, Abidjan, Côte d’Ivoire; 3 IREGE, Université de Savoie, BP 80439, Annecy-le-Vieux, 74944 France

**Keywords:** Health and job causality, Bivariate dynamic probit model, Gauss-Hermite quadrature, I10, J6, C3, C51

## Abstract

This article investigates the causal links between health and employment status. To disentangle correlation from causality effects, the authors leverage a French panel survey to estimate a bivariate dynamic probit model that can account for the persistence effect, initial conditions, and unobserved heterogeneity. The results highlight the crucial role of all three components and reveal strong dual causality between health and employment status. The findings clearly support demands for better coordination between employment and health public policies.

## Background

Health changes and labour market instability both have important impacts on individual well-being, which strongly guide policy makers in defining rules for health insurance, unemployment benefits, and/or retirement. A substantial empirical literature stresses the links between health and labour market risks, yet the precise relationship between the two phenomena remains unclear, leaving the design of appropriate public policies uncertain as well, especially because policies in the labour market can produce health effects (and vice versa).

Early empirical studies focused on one-way causality, such that health conditions explained labour market transitions or *vice versa*. For example, in [1] pioneering study, people’s health determines their labour participation decisions, and [2] confirms that disabilities strongly affect labour participation. As an endowment of human capital, health determines productivity and preferences for work versus leisure [3]. One study by Stewart [4] exhibits the impact of health on the duration of unemployment and one another by Garcia-Gomez et alii [5] shows that health impacts on exits out of and entries into employment. Moreover, two complementary results emerge from a literature review [6]. First, poor health affects everyone’s labour choices, but the impact is especially powerful among the elderly, such that health problems significantly increase choices to retire [7–11], and retirement decisions often represent an attempt to preserve health [12]. Second, the impact of a person’s health varies with the type of health deterioration. Chronic diseases, such as cancer [13], diabetes [14–16], mental illness [17, 18], and disabilities [2] seem to have the strongest effect on individual transitions in the labour market.

In addition, employment status has implications for health. For example, unemployment and inactivity slightly increase the risks of cardiovascular diseases [19], cancer, or mental illnesses [20, 21]. Morris et al. [22] using British data and [23] using Australian data confirm that a loss of employment increases mortality risk. Mesrine [24] shows that this impact is even greater following long spells of unemployment. The pecuniary and non-pecuniary effects of inactivity and unemployment on health help explain these empirical findings. Unemployment usually decreases the health care resources available to the person, so it can affect health over the long-term.

To pursue the topics of causality, some authors do analyze the pathways between the two phenomenons (see Schmitz [25]). For example, unemployment and non-participation in the labour market damage people’s self-esteem [20, 21] and decrease their sense of well-being [26, 27]. Persistent unemployment and inactivity thus create threatening conditions for health. Conversely, being employed can have some deleterious effects on health, such as by increasing the risk of stress, professional illness and work accidents. Thus, [28] uses economic data to argue that bad working conditions and work pain cause damage to people’s health. Using a matching approach with the French Health Survey 2002, [28] shows that workers exposed to poor working conditions consult physicians 25% more than those who are not. Hamon-Cholet and Sandret [29] similarly find, with French data, that noisy jobs increase the professional accident rate to 25%.

However, the links between health and labour status may be more complex than a one-way form of causality. Recently, some authors have emphasized the need to correct for endogeneity between health [16, 30, 31] and labour market transitions [32]. Neglecting endogeneity can cause strong estimate biases. For example, [33] analyses of the European Working Conditions data set indicate that the fear of involuntary job loss has health impacts, such as headaches, eye-strain, and skin problems. Without controlling for the endogeneity of job insecurity, job insecurity degrades all health indicators. This endogeneity of health and job risks likely reflects two main sources. First, unobserved heterogeneity, such as that due to lifestyle or individual preferences, can influence both health and labour market processes [10]. Second, measurement errors in self-reported health surveys or using poor health as a reason to justify unemployment, might create substantial endogeneity biases [34].

Another major source of endogeneity is likely to be reciprocity : Labour activities and health affect each other. Few studies take this simultaneity into account, though [31] propose a bivariate model with a lagged dependent variable to analyze dynamics in health and labour market risk. This approach offers the advantage of addressing endogeneity problems and allowing for a dynamic analysis. Accordingly, these authors show that recent health conditions affect current labour market risk, and vice versa, and that this dynamic is strongly persistent. Such persistence effects also may be due to favorable or unfavorable initial conditions for health and employment [35]. Haan and Myck [36], and Arulampalam and Stewart [31] do not address these potential contingencies. Neglecting these initial conditions could bias estimates of the simultaneity effect between health and employment status. A presentation of the health-employment nexus is available in [38] or in [37].

Finally, we lack clear definitions of all the links between health and job risks. With this article, we propose an innovative methodology for identifying and assessing all the complex links between health and employment paths. With our modeling approach, we can jointly estimate the two phenomena. We assume sequential causality, as in [39, 40] or [31], such that the most recent health status can influence the current labour market status, and the last event in the labour market affects the current period health status. We also account for unobserved heterogeneity and persistence in the two processes over time [41]. Finally, following [42], we control for initial conditions.

As previous empirical work, we aim to establish whether causality exists between health and employment, as well as to define its meaning and scope. In this paper, we make use of the Granger causality framework which assesses only a better predictability for a variable, based on another one. This interpretation of the Granger causality has to be separate from the “cause-effect” relationship concept which is more difficult to assess in social sciences. Nevertheless, it help us to derive insights and guidance for economic policies. If health and employment are independent, policy makers can use disconnected instruments. If single causation exists instead (e.g., job transitions explain health paths but health does not affect job risks), it will be necessary to monitor the effects of an employment-centered policy on health. Finally, if dual causality exists, only the joint design of health and employment policies can improve health and employment simultaneously.

The estimates in this study feature a sample of French individuals who completed the Santé et Itinéraire Professionnel (SIP) survey (DARES (Direction de l’Animation de la Recherche, des Etudes et des Statistiques), the statistical bureau of the French administration for Labor Affairs), DREES (Direction de la Recherche, des Etudes, de l’Evaluation et des Statistiques), the statistical bureau of the French administration for Health Affairs), 2006). This survey (see “Background” section) indicates, for each year since the participant finished school until 2006, all individual events related to health and labour market status. With this long panel data, we can better control for unobserved heterogeneity compared with using cross-sectional data. Moreover, this survey provides empirical evidence of the links between health and labour market paths in France, whereas prior literature has focussed on U.S., British, or Australian data. Significant institutional differences (in terms of legislation regulating the labour market and rules governing health systems) exist across these countries, which limits the generalizability of the results obtained in English-speaking countries to the French case. Focusing on the French case thus might provide new insights and clarify the links between health and labour market transitions, by addressing them in a different kind of health care system.

“Background” section presents the relevant data for this analysis. French longitudinal survey on health and work: SIP” section outlines the innovative methodology we have implemented to investigate the complex links between health and labour market transitions. After we present and discuss the results in “Econometric framework” section, we conclude with some implications and directions for further research.

## French longitudinal survey on health and work: SIP

Conducted in 2006 by DARES and DREES, the Santé et Itinéraire Professionnel (SIP) survey gathered information about 13,991 individuals, aged from 20 to 74 years [43]. This survey describes individual paths on the job market and health status. Each respondent provides the information about previous conditions. The survey data also include socioeconomic information, such as gender, age, grades, income, and ethnicity.

Because we seek to analyze events during people’s professional lives, we exclude those who never entered the job market. We also exclude those who entered before 1962, to observe macroeconomic conditions that may affect individual transitions in the labour market. After data processing, we obtained a sample of 10,569 persons who provided detailed information about their participation in the labour market and their health status, spanning the full professional path of each individual, from the end of schooling to retirement. On average, each respondent thus provides information about a period of 26 years^1^. Pooling the data across all years produces a dataset with 255,206 observations.

For each year of professional life, we distinguish four categories for job status :

- Long time period employments, which last at least five years.
- Short time period employments, which last less than five years.
- Unemployment periods, which last more than one year.
- Out of job market time periods, which last more than one year.

With the first two items, we define all respondents who report being employed in a long-term or short-term job as employed for that given year. Our definition of employed people is thus quite expansive, because non-employment status covers both unemployment and non-participation. In addition, the SIP survey does not offer a means to observe short-term (shorter than one year) unemployment or inactivity. Being employed during a particular year in the survey does not imply that individuals were employed for the entire year though, so measurement errors could arise for the labour market status variable. To avoid this bias, and as robustness tests, we also consider long-term inactivity and unemployment status. These two items also are binary variables, equal to 1 if the respondent is inactive or unemployed for the entire given year.

Moreover, participants self-report whether they have encountered illnesses during a given year. With these data, we can construct a health indicator as a binary variable, equal to 1 if the respondent reports any illness.

Thus, we have two variables: one for health status and one for job status. In the French health care system, level of health insurance is very high. Individuals can receive large benefits from the system when they get any illness. Illness may imply a temporary or permanent job cut. many people in the sample do experience a temporary job cut in case of illness but they resume their job after the end of illness. This imply that some individuals may have the same professional status over time but with some spells of illness.

For a better understanding of health status, we also create a more qualitative indicator, similar to [11]. For each illness reported in the survey, we know the corresponding World Health Organization’s ICD^2^. That code also reveals an indicator of severity and an indicator of disability according to the mapping created by the Institut de Recherche et de Documentation en Économie de la Santé (IRDES). The severity index indicates if the illness is related to a risk of death; the disability index determines if the illness affects the person’s daily life. With this information, we create binary dummy variables to establish whether the risk of death is large (rdeath=1) and whether the disability index is large (disab=1). In turn, we create a percentage measure to reflect the extent to which each situation occurs over the course of the respondent’s full working life.

Because we know the length of each respondent’s professional life, we can calculate synthetic indicators of the professional and health paths: the percentage of professional life with at least one illness and the share of employment, unemployment, and out-of-job market periods in professional life (see Table 1).

**Table 1:** Descriptive statistics for labour market and health paths

Indicators	Means	Std. Err.
Number of years per individual	26.994	12.070
Share of employment periods in professional life	0.863	0.237
Share of unemployment periods in professional life	0.034	0.093
Share of out-of-job market time periods in professional life	0.103	0.219
Share of years with at least one illness in professional life	0.1795	0.295
Share of years with at least one illness with disability	0.028	0.135
Share of years with at least one illness with risk of death	0.019	0.165

Notes: Number of individuals: 10,569

As this table shows (means in column 2 and standard deviations in column 3), employment periods represent a large fraction of the professional life. Only 3.4% of professional life involved long-term unemployment, and 10.3% occurred out of the job market. Illness periods represented almost 18% of the professional life.

Moreover, exploiting the longitudinal dimension of our data, we examine the conditional outcome in period *t*, conditional on the respondents’ self-assessed statuses in the labour market and health in period *t*−1 (Table 2). We find considerable persistence in both the labour market and health paths. For example, conditional on being employed in *t*−1, about 97.8% of respondents report being employed in *t* (on pooled sample).

**Table 2:** Transitions in labour market and health status

	Status at t					
Status at *t*−1	Employed	Unemployed	Out of labour market	Ill	Ill with disability	Ill with risk of death
Employed	0.978	0.011	0.011	0.213	0.028	0.018
Unemployed	0.331	0.622	0.047	0.324	0.036	0.025
Out of labour market	0.081	0.005	0.914	0.289	0.044	0.030
Ill	0.809	0.043	0.148	0.986	0.125	0.082
Ill with disability	0.782	0.039	0.179	0.982	0.982	0.337
Ill with risk of death	0.770	0.043	0.187	0.970	0.512	0.970
Not Ill	0.879	0.022	0.099	0.017	0.003	0.002

Table 1 also presents the labour force status against lagged self-reported health, using the pooled sample. It highlights the negative relationship between poor health and employment. Respondents who declare a disease in *t*−1 are more likely to be unemployed or out of the labour market in *t*. But these statistics also suggest evidence of a reverse link, as suggested in prior literature. Table 1 also contains the health status against the lagged labour market indicators, using the pooled sample. Finally, persistence and simultaneity seem to characterize health and labour market processes.

In addition, some individual attributes can be observed^3^. Table 3 provides the information pertaining to these variables for the pooled sample and for sub-samples defined according the labour market and health status.

**Table 3:** Socioeconomic characteristics

	Employed	Unemployed	Out of labour market	Ill	Ill with		Pooled sample
					Disability	Risk of death	
Men	0.508	0.364	0.095	0.426	0.516	0.481	0.460
Not French ^∗^	0.108	0.141	0.195	0.103	0.059	0.08	0.119
Couple	0.705	0.618	0.808	0.734	0.712	0.634	0.713
Number of children	1.257	1.379	2.020	1.613	1.609	1.561	1.350
No grade	0.068	0.134	0.190	0.089	0.101	0.092	0.084
High School grade	0.537	0.543	0.518	0.536	0.555	0.511	0.534
College grade	0.161	0.162	0.141	0.167	0.175	0.181	0.158
Undergraduate studies	0.095	0.068	0.073	0.083	0.083	0.076	0.092
Graduate studies	0.140	0.093	0.077	0.126	0.087	0.14	0.132
Number of obs.	220,812	8,335	31,817	54,989	7,257	4,830	255,206

^*^Refers to the individual’s nationality

According to these descriptive statistics, persons who do not participate to the labour market in a given year are more likely to have certain specific characteristics. As expected, females, less educated people, and those with children are more likely to be out of the labour market. Conversely, among the employed, we count more men and people with academic degrees. Table 3 also shows that female, French people and those with academic degrees report more numerous illness periods. These statistics do not necessarily mean that respondents suffer poorer health; they might just be more concerned about their health and thus declare more illnesses.

Finally, these descriptive statistics argue for taking simultaneity and persistence effects into account to obtain a robust analysis of causality links between health and employment status. We present an econometric framework to fulfil that goal.

## Econometric framework

### Assessing causality

**Fig. 1:**
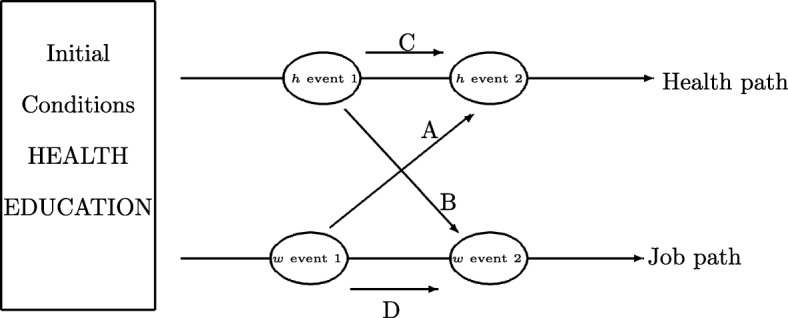
Dynamics of health and job status

We first define two dependent variables: health condition (*h*=1 if an illness is declared, *h*=0 otherwise) and job status (*w*=1 if employed, long or short time periods, *w*=0 otherwise). From the SIP data set, we can observe *h* and *w* for each individual *i* and each year *t*. Thus, we model the interactions between *h*_*it*_ and *w*_*it*_ while accounting for two issues : the path dynamics of each event (and particularly the inertia of each path) and the link between each path. In Fig. 1, we present all the links that may exist between the two events over time.

In the basic example in Fig. 1, four different interactions appear. Links *A* and *B* represent the effect of a health outcome (job status) at time *t*−1 on job status (health outcome) at time *t*. Inertia can also exist (links *C* and *D*, such that the probability of being in a good health condition at time *t*−1 influences the health condition at time *t*). Finally, various sets of control variables may influence *h* and *w*.

To identify all these links clearly, we used the causality concept, introduced by [44]. It defines better predictability for a variable *h* according to the use of its lag values, the lag value of another variable *w*, and some controls *X*. Note that an analogous definition holds for Granger non-causality from *h* to *w*. [44] distinguishes instantaneous causality, such that *w*_*t*_ is causing *h*_*t*_ (if *w*_*t*_ is included in the model, it improves the predictability of *h*_*t*_) from lag causality, in which case the lag values of *w* improve the predictability of *h*_*t*_.

The model specification that we are building considers health status *h* and job status *w* as the main variables. Because of the strong persistence of individual health statuses across time, we do include the lagged value of health to explain current health (Eq. 1). To explain job status and to assess causality, we also use the lagged value of health (Eq. 2). This methodology contrasts with models that use a shock in health status to explain (for example) job status and to assess causality (see [45, 46] or [47]). In these cases and in our paper, the main issue remains the same: is the health variable exogenous? In our model, the bivariate panel data model takes care of the issue.

### Panel data case

Various approaches can be used to test noncausality on panel data. Contrarily to [48] who assume that the causal effects are individual specific, we suppose that the effects are homogeneous among all individuals and at all time. Since *h*_*t*_ and *w*_*t*_ are binary outcome variables, we can use latent variables (*h*^∗^ and *w*^∗^), with the assumption that *h* and *w* have a positive outcomes (equal to 1) if their latent variables are positive.

Then, the model specification is:

1 $$\begin{array}{*{20}l} h_{it} & = & 1 \text{ if}\ h^{*}_{it} >0  \\ & = & 0 \text{ otherwise}  \\ w_{it} & = & 1 \text{ if}\ w^{*}_{it} >0  \\ & = & 0 \text{ otherwise}  \\ h^{*}_{it} & = & X_{it}^{1} \beta_{1} + \delta_{11} h_{i,t-1} + \delta_{12} w_{i,t-1} \\ & & + \eta^{1}_{i} + \zeta^{1}_{it}  \end{array} $$

2 $$\begin{array}{*{20}l} w^{*}_{it} & = & X_{it}^{2} \beta_{2} + \delta_{21} h_{i,t-1} + \delta_{22} w_{i,t-1} \\ & &+ \eta^{2}_{i} + \zeta^{2}_{it}  \end{array} $$

where $\left (\eta ^{1}_{i}, \eta ^{2}_{i}\right)^{\prime }$ denotes the individual random effects that has mean zero and covariance matrix *Σ*_*η*_, and $\left (\zeta ^{1}_{it}, \zeta ^{2}_{it}\right)'$ denotes the idiosyncratic shocks that has mean zero and covariance matrix *Σ*_*ζ*_, with

$$\begin{array}{@{}rcl@{}} \Sigma_{\eta} &=& \left(\begin{array}{ll} \sigma^{2}_{1} & \sigma_{1} \sigma_{2} \rho_{\eta} \\ \sigma_{1} \sigma_{2} \rho_{\eta} & \sigma^{2}_{2} \end{array}\right) \, \\ \Sigma_{\zeta} &=& \left(\begin{array}{ll} 1 & \rho_{\zeta} \\ \rho_{\zeta} & 1 \end{array}\right). \end{array} $$

Testing for Granger noncausality is equivalent to testing *H*0:*δ*_12_=0 for the prediction that *w* is not causing *h* and to testing *H*0:*δ*_21_=0 for the prediction that *h* is not causing *w*.

In our specification, we have ruled out the instantaneous causality as we want to highlight temporarily lagged effects. In our case, looking at instantaneous causality at period *t* is of no interest because of the lack of within year information, as in [49]. In addition, within year job transitions are rather scarce. Within the full population, 86% of individuals have a job at a given time period. More precisely, 68% have a long time period job (more than 5 years) and 18% have a short time period job (less than 5 years).

### Initial conditions

For the first wave of the panel (initial condition), there is a lack of data for the previous state of *h* and *w* (*h*_*i*,0_ and *w*_*i*,0_ are not observed). Thus, *P*(*h*_*i*1_,*w*_*i*1_|*h*_*i*,0_,*w*_*i*,0_,*X*_*i*_) cannot be evaluated. Ignoring it in the individual overall likelihood, implies also ignoring the data generation process for the first wave of the panel. This means that the data generating process of the first wave of the panel is exogenous or in equilibrium. These assumptions hold only if the individual random effects are degenerated. Otherwise, the initial conditions can be explained by the individual random effects, then ignoring them leads to inconsistent parameter estimates [35].

The solution proposed by [35] for the univariate case and generalized to the bivariate case by [39] is to estimate a static equations for the initial conditions. In these static equations, the random effects are a linear combination of the random effects of the dynamic equations. Different idiosyncratic errors terms are specified for the initial conditions. Formally, the initial conditions equations are given by:

3 $$\begin{array}{*{20}l} h^{*}_{i1} & = &Z_{i}^{1} \gamma_{1} + \lambda_{11} \eta^{1}_{i} + \lambda_{12} \eta^{2}_{i}  \\ & & + \epsilon^{1}_{i}  \end{array} $$

4 $$\begin{array}{*{20}l} w^{*}_{i1} & = &Z_{i}^{2} \gamma_{2} + \lambda_{21} \eta^{1}_{i} + \lambda_{22} \eta^{2}_{i}  \\ & & + \epsilon^{2}_{i}  \end{array} $$

where $\left (\begin {array}{l} \epsilon ^{1}_{i} \\ \epsilon ^{2}_{i} \end {array}\right)$ are the idiosyncratic errors with mean zero and covariance matrix $\Sigma _{\epsilon } = \left (\begin {array}{ll} 1 & \rho _{\epsilon } \\ \rho _{\epsilon } & 1 \end {array}\right) $.

Since *η*^1^ and *η*^2^ are individual random effects for *h* and *w*, *λ*_12_ and *λ*_21_ can be interpreted as the effect of *η*^1^ (respectively *η*^2^) on *w* (respectively *h*) for the first wave of the panel.

### Estimation methods for health and job paths

Finally, because we want to estimate the dynamics of health (*h*) and job status (*w*), we set the Eqs. 1 and 2 for each time period (*t*>1) and the Eqs. 3 and 4 for initial conditions.

In Eqs. 1 to 4, many characteristics simultaneously affect health and labour market processes. To achieve the estimations, we also need at least two exclusion restrictions. The variable for the labour market status equation is the national unemployment rate (source: Institut National de Statistiques et d’Etudes Economiques, INSEE). The exclusion restriction for health status is set according to the physician per population ratio, also known as the medical density [50]. Equations 1 and 2 can be consistently estimated under assumption that *η*_*i*_ and *ζ*_*it*_ have symmetric distributions [35]. The individual level likelihood is given by:

$$\begin{array}{@{}rcl@{}} L_{i} & = & \int_{{\mathbb R}^{2}} \Phi_{2}\left(q^{1}_{i0}h_{i}^{0},q^{2}_{i0}w^{0}_{i},q^{1}_{i0}q^{2}_{i0}\rho_{\epsilon}\right) \\ & &\prod_{t=2}^{T_{i}} \;\Phi_{2}\left(q^{1}_{it}\bar{h}_{it},q^{2}_{it}\bar{w}_{it},q^{1}_{it}q^{2}_{it}\rho_{\zeta}\right) \\ & &\phi(\eta_{i},\Sigma_{\eta})d\eta_{i}^{1}d\eta_{i}^{2} \end{array} $$

Where

$$\begin{array}{@{}rcl@{}} q_{it}^{1} & = & 2y^{1}_{it}-1\;\;\forall\;i,t\\  q_{it}^{2} & = & 2y^{2}_{it}-1\;\;\forall\;i,t\\  h_{i}^{0} & = & Z^{1}_{i}\gamma_{1}+\lambda_{11}\eta^{1}_{i}+\lambda_{12}\eta^{2}_{i}\\  w_{i}^{0} & = & Z^{2}_{i}\gamma_{2}+\lambda_{21}\eta_{i}^{1}+\lambda_{22}\eta^{2}_{i}\\  \bar{h}_{it} & = & X^{1}_{it}\beta_{1}+\delta_{11}h_{i,t-1}\\ & & +\delta_{12}w_{i,t-1}+\eta^{1}_{i}\\  \bar{w}_{it} & = &X^{2}_{it}\beta_{2}+\delta_{21}h_{i,t-1}\\ & & +\delta_{22}w_{i,t-1} +\eta^{2}_{i} \\  \end{array} $$

Since the likelihood function has an intractable form, its estimation involves the use of a numerical integration methods. There are two main methods to estimate our likelihood function: the Gauss-Hermite quadrature (GHQ) and the maximum simulated likelihood (MSL). For our estimations, we chose the adaptative Gauss-Hermite quadrature proposed by [51]^4^.

## Results and discussion

We present econometric results in Table 4. In Table 4, columns (1) and (2) contain the results from bivariate probit regressions for Eqs. 1 and 2. In columns (1’) and (2’), we also provide the univariate probit regressions (with no correlation between the two equations) for these equations. We do the same in Table 5 for the initial conditions (Eqs. 3 and 4).

**Table 4:** Estimates of health and job status interactions. Part A: dynamic equations

	Bivariate estimations		Univariate estimations	
*Variables*	*h* : *Illness*	*w* : *work*	*h* : *Illness*	*w* : *work*
	(1)	(2)	(1’)	(2’)
*h*_−1_	$\underset {(0.0225)}{3.8165^{***}}$	$\underset {(0.014)}{-0.2013^{***}}$	$\underset {(0.0154)}{4.2519^{***}}$	$\underset {(0.0158)}{-0.276^{***}}$
*w*_−1_	$\underset {(0.0239)}{0.2193^{***}}$	$\underset {(0.0128)}{2.7189^{***}}$	$\underset {(0.0194)}{0.0242}$	$\underset {(0.0137)}{2.7158^{***}}$
*Age*	$\underset {(0.0056)}{0.0338^{***}}$	$\underset {(0.004)}{0.1079^{***}}$	$\underset {(0.0047)}{-0.0088^{*}}$	$\underset {(0.0042)}{0.1297^{***}}$
*Age square*	$\underset {(0.0001)}{0.00003}$	$\underset {(0.0001)}{-0.0017^{***}}$	$\underset {(0.0001)}{0.0003^{***}}$	$\underset {(0.0001)}{-0.0018^{***}}$
*Not French* ^+^	$\underset {(0.0251)}{-0.0492^{**}}$	$\underset {(0.0166)}{-0.3368^{***}}$	$\underset {(0.0191)}{-0.0226}$	$\underset {(0.0225)}{-0.2434^{***}}$
*Gender*(*male*)	$\underset {(0.0583)}{-0.2814^{***}}$	$\underset {(0.0543)}{0.2843^{***}}$	$\underset {(0.0419)}{-0.081^{*}}$	$\underset {(0.0542)}{-0.0194}$
*Couple*	$\underset {(0.0237)}{-0.0325}$	$\underset {(0.0172)}{-0.482^{***}}$	$\underset {(0.0196)}{-0.0406^{**}}$	$\underset {(0.0185)}{-0.4217^{***}}$
Male * Couple	$\underset {(0.0373)}{0.0391}$	$\underset {(0.0307)}{0.7826^{***}}$	$\underset {(0.0306)}{0.0305}$	$\underset {(0.0324)}{0.6622^{***}}$
*Number of children*	$\underset {(0.0091)}{0.0363^{***}}$	$\underset {(0.0062)}{-0.1554^{***}}$	$\underset {(0.0073)}{0.0278^{***}}$	$\underset {(0.0074)}{-0.1291^{***}}$
Male * Number of children	$\underset {(0.013)}{-0.007}$	$\underset {(0.0106)}{0.0334^{***}}$	$\underset {(0.0104)}{-0.0134}$	$\underset {(0.0116)}{0.0243^{**}}$
*No grade*	$\underset {(0.0492)}{0.2883^{***}}$	$\underset {(0.032)}{-0.7921^{***}}$	$\underset {(0.037)}{0.0844^{**}}$	$\underset {(0.0425)}{-0.5771^{***}}$
*College grade*	$\underset {(0.0345)}{0.2705^{***}}$	$\underset {(0.0252)}{-0.5065^{***}}$	$\underset {(0.0254)}{0.0698^{***}}$	$\underset {(0.0307)}{-0.2658^{***}}$
*High school grade*	$\underset {(0.0401)}{0.2309^{***}}$	$\underset {(0.0293)}{-0.3331^{***}}$	$\underset {(0.0298)}{0.0637^{**}}$	$\underset {(0.0358)}{-0.1739^{***}}$
*Undergraduate studies*	$\underset {(0.0459)}{0.0859^{*}}$	$\underset {(0.034)}{-0.158^{***}}$	$\underset {(0.034)}{0.0218}$	$\underset {(0.0408)}{-0.1139^{***}}$
*Ref : Graduate studies*	-	-	-	-
Male * No grade	$\underset {(0.0755)}{0.2553^{***}}$	$\underset {(0.063)}{-0.1505^{**}}$	$\underset {(0.0556)}{0.0746}$	$\underset {(0.0712)}{0.1912^{***}}$
Male * College grade	$\underset {(0.0556)}{0.1264^{**}}$	$\underset {(0.053)}{-0.0182}$	$\underset {(0.0392)}{0.0402}$	$\underset {(0.0542)}{0.1243^{**}}$
Male * High school grade	$\underset {(0.0663)}{0.0717}$	$\underset {(0.0619)}{-0.0882}$	$\underset {(0.0474)}{0.0378}$	$\underset {(0.065)}{0.0404}$
Male * Undergraduate studies	$\underset {(0.079)}{0.0048}$	$\underset {(0.0789)}{0.1126}$	$\underset {(0.0555)}{0.0139}$	$\underset {(0.0775)}{0.1028}$
Ref : Male * Graduate studies	-	-	-	-
*Medical density*	$\underset {(0.0003)}{-0.0006^{*}}$	−	$\underset {(0.0003)}{0.0022^{***}}$	−
*Unemployment rate*	−	$\underset {(0.0024)}{0.0433^{***}}$	−	$\underset {(0.0026)}{-0.0022}$
*Intercept*	$\underset {(0.0992)}{-2.5964^{***}}$	$\underset {(0.0695)}{-3.1624^{***}}$	$\underset {(0.0827)}{-2.5882^{***}}$	$\underset {(0.072)}{-2.1492^{***}}$
*Covariance matrix*	$\sigma _{1} = \underset {(0.0184)}{1.371^{***}}$, $\sigma _{2} = \underset {(0.0161)}{1.7086^{***}}$		-	
	$\rho _{\eta } = \underset {(0.0054)}{-0.8242^{***}}$, $\rho _{\zeta } = \underset {(0.0176)}{0.0258}$		-	

Standard errors are in parenthesis. ***: Significant at 1% level, **: Significant at 5% level. *: Significant at 10% level, +: Refers to the individual’s nationality

**Table 5:** Estimates of health and job status interactions. Part B: the initial conditions

	Bivariate estimations		Univariate estimations	
*Variables*	*h* : *Illness*	*w* : *work*	*h* : *Illness*	*w* : *work*
*Age*	$\underset {(0.0899)}{-0.0012}$	$\underset {(0.0428)}{0.0806^{*}}$	$\underset {(0.0686)}{0.0221}$	$\underset {(0.0426)}{0.0848^{**}}$
*Age square*	$\underset {(0.0022)}{-0.0001}$	$\underset {(0.001)}{-0.0012}$	$\underset {(0.0017)}{-0.0009}$	$\underset {(0.001)}{-0.0013}$
*Not French* ^+^	$\underset {(0.1022)}{-0.2236^{**}}$	$\underset {(0.0443)}{-0.45^{***}}$	$\underset {(0.078)}{-0.202^{**}}$	$\underset {(0.0442)}{-0.4486^{***}}$
*Gender*(*male*)	$\underset {(0.165)}{-0.1136}$	$\underset {(0.0922)}{-0.3121^{***}}$	$\underset {(0.1215)}{0.0108}$	$\underset {(0.092)}{-0.3264^{***}}$
*Couple*	$\underset {(0.0958)}{-0.0242}$	$\underset {(0.056)}{-0.0243}$	$\underset {(0.0732)}{-0.011}$	$\underset {(0.0558)}{-0.0244}$
Male * Couple	$\underset {(0.1705)}{0.1825}$	$\underset {(0.1051)}{0.519^{***}}$	$\underset {(0.127)}{0.1618}$	$\underset {(0.1051)}{0.5179^{***}}$
*Number of children*	$\underset {(0.164)}{-0.0384}$	$\underset {(0.0753)}{-0.6685^{***}}$	$\underset {(0.122)}{0.0338}$	$\underset {(0.0752)}{-0.6732^{***}}$
Male * Number of children	$\underset {(0.2873)}{0.0417}$	$\underset {(0.1456)}{0.4619^{***}}$	$\underset {(0.2071)}{-0.0673}$	$\underset {(0.1454)}{0.4731^{***}}$
*No grade*	$\underset {(0.1991)}{0.2148}$	$\underset {(0.1028)}{-1.035^{***}}$	$\underset {(0.1539)}{0.1209}$	$\underset {(0.1025)}{-1.0383^{***}}$
*College grade*	$\underset {(0.1384)}{0.1299}$	$\underset {(0.0788)}{-0.3124^{***}}$	$\underset {(0.1041)}{0.0861}$	$\underset {(0.0787)}{-0.3074^{***}}$
*High school grade*	$\underset {(0.1421)}{0.1154}$	$\underset {(0.0807)}{-0.2995^{***}}$	$\underset {(0.1076)}{0.0476}$	$\underset {(0.0806)}{-0.2958^{***}}$
*Undergraduate studies*	$\underset {(0.1448)}{0.062}$	$\underset {(0.0932)}{0.1695^{*}}$	$\underset {(0.1085)}{0.0797}$	$\underset {(0.0931)}{0.1698^{*}}$
*Ref : Graduate studies*	-	-	-	-
Male * No grade	$\underset {(0.2647)}{-0.0197}$	$\underset {(0.1366)}{1.1132^{***}}$	$\underset {(0.2025)}{-0.1556}$	$\underset {(0.1361)}{1.1343^{***}}$
Male * College grade	$\underset {(0.1792)}{-0.1164}$	$\underset {(0.0998)}{0.5157^{***}}$	$\underset {(0.1331)}{-0.2098}$	$\underset {(0.0997)}{0.5252^{***}}$
Male * High school grade	$\underset {(0.2178)}{-0.4229^{*}}$	$\underset {(0.1134)}{0.1922^{*}}$	$\underset {(0.1615)}{-0.3716^{**}}$	$\underset {(0.1133)}{0.2008^{*}}$
Male * Undergraduate studies	$\underset {(0.2403)}{-0.3887}$	$\underset {(0.1317)}{-0.331^{**}}$	$\underset {(0.1759)}{-0.3672^{**}}$	$\underset {(0.1316)}{-0.3314^{**}}$
Ref : Male * Graduate studies	-	-	-	-
*Medical density*	$\underset {(0.0008)}{0.0009}$	−	$\underset {(0.0006)}{0.0027^{***}}$	−
*Unemployment rate*	−	$\underset {(0.0048)}{0.0001}$	−	$\underset {(0.0046)}{-0.0031}$
*Illness before professional life*	$\underset {(0.0124)}{0.3626^{***}}$	$\underset {(0.0048)}{-0.0023}$	$\underset {(0.009)}{0.3473^{***}}$	$\underset {(0.0045)}{-0.0028}$
*Intercept*	$\underset {(0.9148)}{-1.693^{*}}$	$\underset {(0.4399)}{0.217}$	$\underset {(0.704)}{-2.2141^{***}}$	$\underset {(0.4377)}{0.2683}$
*λ*_11_	$\underset {(0.0633)}{1.2011^{***}}$	-		
*λ*_12_	$\underset {(0.0567)}{0.3897^{***}}$	-		
*λ*_21_	-	$\underset {(0.0296)}{0.0233}$		
*λ*_22_	-	$\underset {(0.0264)}{0.0737^{***}}$		
*Covariance matrix*	$\rho _{\epsilon } = \underset {(0.0461)}{0.0185}$		-	

Standard errors are in parenthesis. ***: Significant at 1% level, **: Significant at 5% level. *: Significant at 10% level, +: Refers to the individual’s nationality

The results clearly reveal persistence effects in the health (*δ*_11_=3.8165) and employment (*δ*_22_=2.7189) paths. As [31] suggest, we thus confirm the need to study these phenomena dynamically to explain the situation for each individual in terms of her or his health and employment at time *t*. Evidence for persistence effects also comes from the influence of initial conditions, which depend on various covariates (see Table 5).

As in Hernãndez-Quevedo et alii [52], we pay attention to the effects of individual socio-economic characteristics. We find the expected and well-known effects of socio economic variables on initial health and employment status. Men are less likely to declare an illness and to be employed than women. Elderly people have worse health and job statuses than young people. People without French nationality report less illness and poorer job statuses. Family life also affects health and job conditions: Living as a couple lowers the probability of illness and job stability. The more children in the household, the more illness people experience, and the worse job conditions. Education level creates big differences. More educated people have a lower probability of illness and more likely to be employed. We include some interaction terms between gender and some others socio economic variables to account for gender discrimination in terms of health and job statuses. We find that for men, living as a couple increases the probability of job stability but has no significant effect on the probability to report illness. Men with higher school grade are less likely to report illness than women. For initial condition, we also find that for men, living in couple and having an higher number of children increase the probability to enter job market. However, the higher the school grade for men at the initial state, the lower the probability to enter job market.

The main focus of this paper is on the causality between health and employment status. The bivariate estimates in Table 4 offer strong support. The impact of job status on health is reflected by the coefficient *δ*_12_=0.2288, such that people who have a job at time *t*−1 are more likely to report an illness in the next period *t* (with an increase of 0.0291 in probability to report an illness at *t*, see Table 6 for marginal effects). Two factors could explain these results. First, it could highlight a job quality effect. If being employed involves poor conditions, employment status could readily increase the probability of illness, as argued by [28]. Unfortunately the SIP survey does not identify longitudinal job quality, so we cannot identify the distinct effect of good or poor working conditions. Second, in France, the health care and insurance system is generous for employed people. For example, they may make regular appointments with their physician, which gives them access to more efficient health monitoring. As a result, they may be more likely to detect and report a disease.

Reporting an illness at time *t*−1 lowers the probability of having a job at time *t* (*δ*_21_=−0.1927). The marginal effect of illness on the probability of having a job is − 0.039. This result illustrates that an illness often makes it difficult to stay in a job or to find a new job [6]. Our main contribution is thus to conclude that health and employment status do not have a one way causality path but instead show a dual causality effect^5^.

This result derives from taking into account three sources of bias, as described in “Econometric framework” section: persistence effects, initial conditions, and unobserved heterogeneity. If all these biases were neglected, as in univariate probit models (columns (1’) and (2) of Table 4), estimates of the causality effects between health and employment status would be biased. In our case, we would have wrongly concluded that being employed in previous year has no effect on health.

Finally, the existence of the causality between health and employment status also appears evident in Table 5. The coefficients *λ*_11_ and *λ*_22_ are both significant, confirming the need to integrate unobserved individual effects *η* in our model. In addition, the coefficient *λ*_12_>0 shows that the unobserved individual effect explaining job status (*η*^2^) influences the value of health status at time *t*=1. The method we have developed here is based on the existence of a correlation between unobservable variables in Eqs. 1 and 2 and those of Eqs. 3 and 4. Table 4 gives the values of these correlations. In equations for time *t*>1 and the initial conditions, correlations between idiosyncratic components are not significant. Therefore, the main unobserved heterogeneity, responsible for the correlation, can be captured with individual-specific effects. In the main equations (*t*>1), the correlation between individual-specific effects is negative. Therefore, we call for bivariate panel models to avoid any bias in the estimates. We also establish that individual unobserved factors that explain the probability of having a job (*w*=1) are negatively correlated (*ρ*_*η*_=−0.8242 Table 4) with individual unobserved factors that explain the probability of declaring an illness (*h*=1). Among these unobserved factors, individual intrinsic motivation to job and job satisfaction appear to influence individuals’ health^6^.

Taking advantage of the others two indicators of illness (risk of death and disability, Table 2 for descriptive statistics), we provide the estimation results with these variables in Tables 12, 13, 14 and 15. Using these two additional measures of self-reported illnesses gives support to our main results even if we cannot evaluate the bias in our measures [53]. Table 12 contains the bivariate results for the indicator of disability and Table 14 contains the bivariate results for the indicator of risk of death. The causality from poor health to job status is confirmed to be significant by the coefficients *δ*_21_=−0.4482 for the disability index and *δ*_21_=−0.4876 for the risk of death. Turning to marginal effects, we find that the same and even a stronger effect of health status on the probability of having a good quality job emerges, compared with the previous health indicator (the marginal effects are − 0.106 for the risk of death indicator see Table 8; and − 0.1185 for the indicator of disability, see Table 10). When looking at the impact of job status on health, in contrast with our prior result, we find a weakly significant causal effect of job on illness with disability and no significant effect of job on illness with risk of death. We offer two possible interpretations: First, good jobs provide access to better health coverage and increase the probability of reporting an illness (of any kind). Second, having a job is correlated with poor working conditions. When we control for the severity of health conditions, we find additional support for the first interpretation. Even if people appear induced to report an illness when they have a good job and insurance coverage, the illnesses they report are not particularly severe. We find that the marginal effects (see Tables 6, 7, 8, 9, 10 and 11) of having job on the probability to report an illness high disability index is significant (0.0006) while this effect is not significant (0.0003) on the probability to report an illness with risk of death, contrarily to illness regardless the degree of severity 0.0842. Tables 12 and 14 provide estimated coefficients for our two indicators of illness that account for interaction between gender and others socio economic variables. We find that the higher school grade effect for men does not remain significant. We also find a weak evidence that men with high number of children are less likely to report an illness with risk of disability than women. In terms of initial conditions, there is no discrimination between men and women for the probability of reporting any king of illness.

As with the main health indicator (Table 4), we find a significant correlation between individual-specific effects of health and the job status equations (*ρ*_*η*_=−0.628, Table 12 and *ρ*_*η*_=−0.691, Table 14). The interpretation of the negative sign of these correlations is rather complex. Some unobservable factors that explain the probability of having a job and severe health conditions simultaneously also correlate negatively, such as the existence of specific policies designed to protect the job status of disabled persons.

Finally, and contrary to ([31], see page 1124) claim that “accounting for unobserved heterogeneity reduces the magnitude of the estimated coefficients on the lagged endogenous variables and significantly reduces the persistence of both processes”, our estimates clearly show that causality links (A and B, Fig. 1) are rather strong, regardless of the illness severity.

## Conclusions

This article has examined the relationship between health and labour market paths. As some econometric results fail to account for all the links between health and job market status and thus cannot assess any relationship, we propose a method based on a bivariate dynamic probit model that acknowledges the simultaneity effects between the two phenomena, persistence effects, the role of the initial conditions, and the influence of unobserved heterogeneity. Using a French longitudinal survey we analyze complex interlinks between past and current levels of health and labour market paths. Our results regarding the causality between our two economic outcomes are innovative, due to the econometric methodology and the data set we use.

We demonstrate persistence in both processes. Being ill at *t*−1 is a significant determinant of current health status. Simultaneously, we observe the same persistence in labour market paths. We also confirm the impact of initial conditions, which depend on an individual attributes and macroeconomic conditions.

Taking advantage of this original econometric modeling, which allows us to distinguish between correlation and causality effects, we highlight some significant causal effects between employment and health processes. Being ill at *t*−1 is a significant determinant of current labour market status, and lagged employment has a significant effect on the probability of being ill at time *t*. In addition, we find an influence of unobserved heterogeneity on the causality effects. These effects are strengthened by the existence of individual-specific effects, which are correlated. When taking these effects into account in our bivariate model, we avoid many biases that univariate modeling cannot avoid. In other words, studies who fail to take into account (i) the bidirectional causality between health and employment and (ii) the correlation between unobservables related to health and employment, are not relevant to argue.

As a consequence, our econometric methodology gives us robust estimates of the complex links between health and employment status. As, in our study, we make use of a French data set, we cannot generalize our results to other countries, as can our methodology. Our results therefore argue for a joint design, in France, of health and employment public policies taking interactions between health and employment into account.

## Appendix

**Table 6:** Estimated marginal effects on joint and marginal probabilities. Part A: dynamic equations

*Variables*	*p*(*h*=1,*w*=1)	*p*(*h*=1)	*p*(*w*=1)
*h*_−1_	$\underset {(0.0034)}{0.5924^{***}}$	$\underset {(0.0022)}{0.8748^{***}}$	$\underset {(0.0032)}{-0.039^{***}}$
*w*_−1_	$\underset {(0.0015)}{0.2101^{***}}$	$\underset {(0.0028)}{0.0291^{***}}$	$\underset {(0.0027)}{0.7216^{***}}$
*Age*	$\underset {(0.0001)}{0.002^{***}}$	$\underset {(0.0002)}{0.0049^{***}}$	$\underset {(0.0002)}{-0.0018^{***}}$
*Not French* ^+^	$\underset {(0.0028)}{-0.0272^{***}}$	$\underset {(0.0034)}{-0.0066^{*}}$	$\underset {(0.004)}{-0.0707^{***}}$
*Gender*(*male*)	$\underset {(0.002)}{0.0435^{***}}$	$\underset {(0.0024)}{-0.022^{***}}$	$\underset {(0.0029)}{0.1805^{***}}$
*Couple*	$\underset {(0.0022)}{-0.0152^{***}}$	$\underset {(0.0026)}{-0.0017}$	$\underset {(0.0028)}{-0.0423^{***}}$
*Number of children*	$\underset {(0.0008)}{-0.0059^{***}}$	$\underset {(0.001)}{0.0046^{***}}$	$\underset {(0.0011)}{-0.0282^{***}}$
*No grade*	$\underset {(0.0041)}{-0.0154^{***}}$	$\underset {(0.0052)}{0.0502^{***}}$	$\underset {(0.0059)}{-0.1699^{***}}$
*College grade*	$\underset {(0.0026)}{0.0018}$	$\underset {(0.0031)}{0.0397^{***}}$	$\underset {(0.0036)}{-0.0906^{***}}$
*High school grade*	$\underset {(0.0033)}{0.0046}$	$\underset {(0.0039)}{0.0303^{***}}$	$\underset {(0.0044)}{-0.06^{***}}$
*Undergraduate studies*	$\underset {(0.0036)}{0.0011}$	$\underset {(0.0042)}{0.0092^{**}}$	$\underset {(0.0048)}{-0.0185^{***}}$
*Ref* : *Graduate studies*	-	-	-
*Medical density*	$\underset {(0.00003)}{-0.0001^{**}}$	$\underset {(0.00005)}{-0.0001^{**}}$	−
*Unemployment rate*	$\underset {(0.0085)}{0.0026}$	−	$\underset {(0.0267)}{0.0081}$

Standard errors are in parenthesis; ***: Significant at 1% level; **: Significant at 5% level; *: Significant at 10% level. +: Refers to individual’s nationality; *h* : *Illness*; *w* : *work*

**Table 7:** Estimated marginal effects on joint and marginal probabilities. Part B: initial conditions

*Variables*	*p*(*h*=1,*w*=1)	*p*(*h*=1)	*p*(*w*=1)
*Age*	$\underset {(0.0026)}{0.0272^{***}}$	$\underset {(0.0027)}{-0.0002}$	$\underset {(0.008)}{0.2475^{***}}$
*Not French* ^+^	$\underset {(0.0066)}{-0.0163^{**}}$	$\underset {(0.0078)}{-0.0195^{**}}$	$\underset {(0.0104)}{-0.0003}$
*Gender*(*male*)	$\underset {(0.0054)}{-0.0161^{***}}$	$\underset {(0.0065)}{-0.0233^{***}}$	$\underset {(0.008)}{0.0447^{***}}$
*Couple*	$\underset {(0.0067)}{-0.0048}$	$\underset {(0.0084)}{0.0045}$	$\underset {(0.0145)}{-0.0819^{***}}$
*Number of children*	$\underset {(0.0111)}{0.0053}$	$\underset {(0.013)}{-0.002}$	$\underset {(0.0167)}{0.0537^{***}}$
*No grade*	$\underset {(0.017)}{0.029^{*}}$	$\underset {(0.0183)}{0.0228}$	$\underset {(0.013)}{0.0765^{***}}$
*College grade*	$\underset {(0.0097)}{-0.0058}$	$\underset {(0.0115)}{0.0104}$	$\underset {(0.015)}{-0.1142^{***}}$
*High school grade*	$\underset {(0.0094)}{-0.0037}$	$\underset {(0.0107)}{-0.0027}$	$\underset {(0.0127)}{-0.0063}$
*Undergraduate studies*	$\underset {(0.009)}{-0.008}$	$\underset {(0.0102)}{-0.0052}$	$\underset {(0.0143)}{-0.038^{***}}$
*Ref* : *Graduate studies*	-	-	-
*Medical density*	$\underset {(0.0001)}{0.00005}$	$\underset {(0.0001)}{0.0001}$	−
*Unemployment rate*	$\underset {(0.0006)}{-0.0081^{***}}$	−	$\underset {(0.0023)}{-0.0771^{***}}$
*Illness before prof. life*	$\underset {(0.003)}{0.0297^{***}}$	$\underset {(0.0037)}{0.0354^{***}}$	$\underset {(0.0016)}{0.0002}$

Standard errors are in parenthesis; ***: Significant at 1% level; **: Significant at 5% level; *: Significant at 10% level. +: Refers to individual’s nationality; *h* : *Illness*; *w* : *work*

**Table 8:** Estimated marginal effects on joint and marginal probabilities. Part A: dynamic equations

*Variables*	*p*(*h*=1,*w*=1)	*p*(*h*=1)	*p*(*w*=1)
*h*_−1_	$\underset {(0.0084)}{0.5725^{***}}$	$\underset {(0.0015)}{0.9827^{***}}$	$\underset {(0.0086)}{-0.106^{***}}$
*w*_−1_	$\underset {(0.0003)}{0.0176^{***}}$	$\underset {(0.0003)}{0.0006^{*}}$	$\underset {(0.0025)}{0.6821^{***}}$
*Age*	$\underset {(0.00001)}{-0.0001^{***}}$	$\underset {(0.00002)}{0.0001^{***}}$	$\underset {(0.0002)}{-0.0021^{***}}$
*Not French* ^+^	$\underset {(0.0003)}{-0.0028^{***}}$	$\underset {(0.0003)}{-0.0008^{**}}$	$\underset {(0.0042)}{-0.072^{***}}$
*Gender*(*male*)	$\underset {(0.0002)}{0.0077^{***}}$	$\underset {(0.0002)}{0.0005^{*}}$	$\underset {(0.003)}{0.211^{***}}$
*Couple*	$\underset {(0.0002)}{-0.0007^{***}}$	$\underset {(0.0003)}{0.0001}$	$\underset {(0.003)}{-0.0421^{***}}$
*Number of children*	$\underset {(0.0001)}{-0.0009^{***}}$	$\underset {(0.0001)}{0.0001}$	$\underset {(0.0012)}{-0.0314^{***}}$
*No grade*	$\underset {(0.0004)}{-0.0056^{***}}$	$\underset {(0.0005)}{0.0013^{**}}$	$\underset {(0.0063)}{-0.1954^{***}}$
*College grade*	$\underset {(0.0003)}{-0.003^{***}}$	$\underset {(0.0003)}{0.0008^{**}}$	$\underset {(0.0039)}{-0.1052^{***}}$
*High school grade*	$\underset {(0.0004)}{-0.0016^{***}}$	$\underset {(0.0004)}{0.0012^{***}}$	$\underset {(0.0048)}{-0.07^{***}}$
*Undergraduate studies*	$\underset {(0.0004)}{0.0018^{***}}$	$\underset {(0.0005)}{0.0004}$	$\underset {(0.0047)}{0.006}$
*Ref : Graduate studies*	-	-	-
*Medical density*	$\underset {(0.00004)}{0.00001}$	$\underset {(0.0001)}{0.00002}$	−
*Unemployment rate*	$\underset {(0.0005)}{0.0003}$	−	$\underset {(0.0251)}{0.009}$

Standard errors are in parenthesis; ***: Significant at 1% level; **: Significant at 5% level; *: Significant at 10% level. +: Refers to individual’s nationality; *h* : *Ill with disability*; *w* : *work*

**Table 9:** Estimated marginal effects on joint and marginal probabilities. Part B: initial conditions

*Variables*	*p*(*h*=1,*w*=1)	*p*(*h*=1)	*p*(*w*=1)
*Age*	$\underset {(0.0005)}{-0.001^{**}}$	$\underset {(0.0005)}{0.0005}$	$\underset {(0.0052)}{-0.1931^{***}}$
*Not French* ^+^	$\underset {(0.0014)}{-0.0045^{***}}$	$\underset {(0.0018)}{-0.0058^{***}}$	$\underset {(0.0111)}{-0.0003}$
*Gender*(*male*)	$\underset {(0.0012)}{0.0011}$	$\underset {(0.0015)}{0.001}$	$\underset {(0.0086)}{0.0414^{***}}$
*Couple*	$\underset {(0.0014)}{-0.0032^{**}}$	$\underset {(0.0018)}{-0.0034^{*}}$	$\underset {(0.015)}{-0.0796^{***}}$
*Number of children*	$\underset {(27.8337)}{-0.0326}$	$\underset {(34.2022)}{-0.0405}$	$\underset {(0.1046)}{0.056}$
*No grade*	$\underset {(0.0056)}{0.0122^{**}}$	$\underset {(0.006)}{0.0128^{**}}$	$\underset {(0.0141)}{0.0816^{***}}$
*College grade*	$\underset {(0.0019)}{0.0034^{*}}$	$\underset {(0.0026)}{0.0054^{**}}$	$\underset {(0.0158)}{-0.1165^{***}}$
*High school grade*	$\underset {(0.0018)}{0.0021}$	$\underset {(0.0022)}{0.0026}$	$\underset {(0.0137)}{-0.0058}$
*Undergraduate studies*	$\underset {(0.0019)}{0.0031}$	$\underset {(0.0024)}{0.004^{*}}$	$\underset {(0.0154)}{-0.0384^{**}}$
*Ref : Graduate studies*	-	-	-
*Medical density*	$\underset {(0.0002)}{0.00004}$	$\underset {(0.0002)}{0.00004}$	−
*Unemployment rate*	$\underset {(0.0001)}{-0.0005^{***}}$	−	$\underset {(0.0018)}{-0.0559^{***}}$
*Illness before prof. life*	$\underset {(0.0002)}{0.0011^{***}}$	$\underset {(0.0003)}{0.0014^{***}}$	$\underset {(0.0011)}{-0.0012}$

Standard errors are in parenthesis; ***: Significant at 1% level; **: Significant at 5% level; *: Significant at 10% level. +: Refers to individual’s nationality; *h* : *Ill with disability*; *w* : *work*

**Table 10:** Estimated marginal effects on joint and marginal probabilities. Part A: dynamic equations

*Variables*	*p*(*h*=1,*w*=1)	*p*(*h*=1)	*p*(*w*=1)
*h*_−1_	$\underset {(0.01)}{0.5527^{***}}$	$\underset {(0.0022)}{0.9741^{***}}$	$\underset {(0.0102)}{-0.1185^{***}}$
*w*_−1_	$\underset {(0.0002)}{0.0121^{***}}$	$\underset {(0.0003)}{0.0003}$	$\underset {(0.0025)}{0.7034^{***}}$
*Age*	$\underset {(0.00001)}{-0.0001^{***}}$	$\underset {(0.00002)}{0.0001^{***}}$	$\underset {(0.0002)}{-0.0022^{***}}$
*Not French* ^+^	$\underset {(0.0002)}{-0.0021^{***}}$	$\underset {(0.0003)}{-0.0006^{*}}$	$\underset {(0.0042)}{-0.0743^{***}}$
*Gender*(*male*)	$\underset {(0.0002)}{0.0045^{***}}$	$\underset {(0.0002)}{0.00003}$	$\underset {(0.003)}{0.2002^{***}}$
*Couple*	$\underset {(0.0002)}{-0.0012^{***}}$	$\underset {(0.0003)}{-0.0007^{**}}$	$\underset {(0.0031)}{-0.0408^{***}}$
*Number of children*	$\underset {(0.0001)}{-0.0007^{***}}$	$\underset {(0.0001)}{0.0001}$	$\underset {(0.0012)}{-0.0315^{***}}$
*No grade*	$\underset {(0.0004)}{-0.0041^{***}}$	$\underset {(0.0005)}{0.0009^{*}}$	$\underset {(0.0063)}{-0.1951^{***}}$
*College grade*	$\underset {(0.0003)}{-0.0025^{***}}$	$\underset {(0.0003)}{0.0002}$	$\underset {(0.0039)}{-0.1055^{***}}$
*High school grade*	$\underset {(0.0003)}{-0.0012^{***}}$	$\underset {(0.0004)}{0.0008^{*}}$	$\underset {(0.0048)}{-0.0701^{***}}$
*Undergraduate studies*	$\underset {(0.0004)}{-0.0006}$	$\underset {(0.0005)}{0.0001}$	$\underset {(0.0053)}{-0.0247^{***}}$
*Ref : Graduate studies*	-	-	-
*Medical density*	$\underset {(0.000004)}{0.000006}$	$\underset {(0.000005)}{0.000009}$	−
*Unemployment rate*	$\underset {(0.0003)}{0.0002}$	−	$\underset {(0.0264)}{0.0092}$

Standard errors are in parenthesis; ***: Significant at 1% level; **: Significant at 5% level; *: Significant at 10% level. +: Refers to individual’s nationality; *h* : *Illness with risk of death*; *w* : *work*

**Table 11:** Estimated marginal effects on joint and marginal probabilities. Part B: initial conditions

*Variables*	*p*(*h*=1,*w*=1)	*p*(*h*=1)	*p*(*w*=1)
*Age*	$\underset {(0.0006)}{-0.0018^{***}}$	$\underset {(0.0006)}{0.0001}$	$\underset {(0.0108)}{-0.3955^{***}}$
*Not French* ^+^	$\underset {(0.001)}{-0.0029^{***}}$	$\underset {(0.0013)}{-0.0038^{***}}$	$\underset {(0.0112)}{-0.0003}$
*Gender*(*male*)	$\underset {(0.0009)}{-0.0001}$	$\underset {(0.0012)}{-0.0005}$	$\underset {(0.0085)}{0.0419^{***}}$
*Couple*	$\underset {(0.0013)}{0.0002}$	$\underset {(0.0017)}{0.0008}$	$\underset {(0.0152)}{-0.0835^{***}}$
*Number of children*	$\underset {(0.6768)}{-0.0139}$	$\underset {(0.8238)}{-0.0174}$	$\underset {(0.0586)}{0.0524}$
*No grade*	$\underset {(0.0042)}{0.0054}$	$\underset {(0.0046)}{0.0054}$	$\underset {(0.0145)}{0.0836^{***}}$
*College grade*	$\underset {(0.0017)}{-0.0001}$	$\underset {(0.0022)}{0.0007}$	$\underset {(0.0161)}{-0.1132^{***}}$
*High school grade*	$\underset {(0.0016)}{-0.0006}$	$\underset {(0.002)}{-0.0007}$	$\underset {(0.0139)}{-0.0053}$
*Undergraduate studies*	$\underset {(0.0015)}{-0.0009}$	$\underset {(0.0019)}{-0.0009}$	$\underset {(0.0156)}{-0.0386^{**}}$
*Ref : Graduate studies*	-	-	-
*Medical density*	$\underset {(0.0001)}{0.00002}$	$\underset {(0.0002)}{0.00003}$	−
*Unemployment rate*	$\underset {(0.0001)}{-0.0004^{***}}$	−	$\underset {(0.0024)}{-0.0838^{***}}$
*Illness before prof. life*	$\underset {(0.0002)}{0.0007^{***}}$	$\underset {(0.0002)}{0.0009^{***}}$	$\underset {(0.0011)}{-0.0016}$

Standard errors are in parenthesis; ***: Significant at 1% level; **: Significant at 5% level; *: Significant at 10% level. +: Refers to individual’s nationality; *h* : *Illness with risk of death*; *w* : *work*

**Table 12:** Estimates of health and job status interactions. Part A: dynamic equations

	Bivariate estimations		Univariate estimations	
*Variables*	*h* : *disab*	*w* : *work*	*h* : *disab*	*w* : *work*
	(1)	(2)	(1’)	(2’)
*h*_−1_	$\underset {(0.0408)}{5.0102^{***}}$	$\underset {(0.033)}{-0.4482^{***}}$	$\underset {(0.0382)}{4.9044^{***}}$	$\underset {(0.0373)}{-0.3705^{***}}$
*w*_−1_	$\underset {(0.0418)}{0.0709^{*}}$	$\underset {(0.0129)}{2.7106^{***}}$	$\underset {(0.041)}{0.034}$	$\underset {(0.0137)}{2.716^{***}}$
*Age*	$\underset {(0.0103)}{0.0067}$	$\underset {(0.004)}{0.109^{***}}$	$\underset {(0.0102)}{0.0111}$	$\underset {(0.0042)}{0.1291^{***}}$
*Age square*	$\underset {(0.0001)}{-0.000002}$	$\underset {(0.0001)}{-0.0017^{***}}$	$\underset {(0.0001)}{-0.00005}$	$\underset {(0.0001)}{-0.0018^{***}}$
*Not French* ^+^	$\underset {(0.045)}{-0.0934^{**}}$	$\underset {(0.0167)}{-0.3174^{***}}$	$\underset {(0.0444)}{-0.099^{**}}$	$\underset {(0.0226)}{-0.2362^{***}}$
*Gender*(*male*)	$\underset {(0.0974)}{-0.0157}$	$\underset {(0.0585)}{0.3627^{***}}$	$\underset {(0.0946)}{0.0176}$	$\underset {(0.0543)}{-0.0036}$
*Couple*	$\underset {(0.0444)}{0.0061}$	$\underset {(0.0173)}{-0.4767^{***}}$	$\underset {(0.0435)}{-0.0048}$	$\underset {(0.0185)}{-0.4119^{***}}$
Male * Couple	$\underset {(0.065)}{0.0083}$	$\underset {(0.031)}{0.7711^{***}}$	$\underset {(0.0642)}{0.0127}$	$\underset {(0.0323)}{0.6558^{***}}$
*Number of children*	$\underset {(0.0155)}{0.0249}$	$\underset {(0.0063)}{-0.159^{***}}$	$\underset {(0.0154)}{0.0231}$	$\underset {(0.0074)}{-0.1291^{***}}$
Male * Number of children	$\underset {(0.0216)}{-0.0215}$	$\underset {(0.0108)}{0.0306^{***}}$	$\underset {(0.0216)}{-0.0294}$	$\underset {(0.0116)}{0.0239^{**}}$
*No grade*	$\underset {(0.0855)}{0.0599}$	$\underset {(0.0324)}{-0.8122^{***}}$	$\underset {(0.0837)}{0.1107}$	$\underset {(0.0427)}{-0.5876^{***}}$
*College grade*	$\underset {(0.0602)}{0.0401}$	$\underset {(0.0255)}{-0.5298^{***}}$	$\underset {(0.0594)}{0.0655}$	$\underset {(0.0308)}{-0.2733^{***}}$
*High school grade*	$\underset {(0.0676)}{0.1093}$	$\underset {(0.0296)}{-0.3557^{***}}$	$\underset {(0.0675)}{0.1174^{*}}$	$\underset {(0.0359)}{-0.1829^{***}}$
*Undergraduate studies*	$\underset {(0.0787)}{0.0507}$	$\underset {(0.0346)}{-0.1874^{***}}$	$\underset {(0.0775)}{0.0558}$	$\underset {(0.041)}{-0.1138^{***}}$
*Ref : Graduate studies*	-	-	-	-
Male * No grade	$\underset {(0.1217)}{0.1847}$	$\underset {(0.0663)}{-0.2431^{***}}$	$\underset {(0.119)}{0.1406}$	$\underset {(0.0714)}{0.1908^{***}}$
Male * College grade	$\underset {(0.0911)}{0.1229}$	$\underset {(0.0568)}{-0.0628}$	$\underset {(0.0886)}{0.0923}$	$\underset {(0.0544)}{0.1234^{**}}$
Male * High school grade	$\underset {(0.1045)}{0.0707}$	$\underset {(0.0656)}{-0.1028}$	$\underset {(0.1028)}{0.0649}$	$\underset {(0.0652)}{0.0414}$
Male * Undergraduate studies	$\underset {(0.1246)}{-0.004}$	$\underset {(0.0824)}{2.0291^{***}}$	$\underset {(0.1217)}{0.0371}$	$\underset {(0.0776)}{0.0984}$
Ref : Male * Graduate studies	-	-	-	-
*Medical density*	$\underset {(0.0006)}{0.0021^{***}}$	−	$\underset {(0.0006)}{0.0015^{**}}$	−
*Unemployment rate*	−	$\underset {(0.0024)}{0.0424^{***}}$	−	$\underset {(0.0026)}{-0.003}$
*Intercept*	$\underset {(0.1858)}{-3.5654^{***}}$	$\underset {(0.07)}{-3.1519^{***}}$	$\underset {(0.1846)}{-3.6125^{***}}$	$\underset {(0.072)}{-2.1525^{***}}$
*Covariance matrix*	$\sigma _{1} = \underset {(0.0013)}{0.1606^{***}}$, $\sigma _{2} = \underset {(0.016)}{1.6948^{***}}$		-	
	$\rho _{\eta } = \underset {(0.0072)}{-0.628^{***}}$, $\rho _{\zeta } = \underset {(0.0387)}{0.0337}$		-	

Standard errors are in parenthesis. ***: Significant at 1% level, **: Significant at 5% level. *: Significant at 10% level, +: Refers to the individual’s nationality

**Table 13:** Estimates of health and job status interactions. Part B: the initial conditions

	Bivariate estimations		Univariate estimations	
*Variables*	*h* : *disab*	*w* : *work*	*h* : *disab*	*w* : *work*
*Age*	$\underset {(0.147)}{-0.002}$	$\underset {(0.0432)}{0.0786^{*}}$	$\underset {(0.1435)}{0.0212}$	$\underset {(0.0426)}{0.0848^{**}}$
*Age square*	$\underset {(0.0035)}{0.0007}$	$\underset {(0.0011)}{-0.0011}$	$\underset {(0.0034)}{0.0001}$	$\underset {(0.001)}{-0.0013}$
*Not French* ^+^	$\underset {(0.2391)}{-0.535^{**}}$	$\underset {(0.0446)}{-0.4658^{***}}$	$\underset {(0.236)}{-0.5691^{**}}$	$\underset {(0.0442)}{-0.4486^{***}}$
*Gender*(*male*)	$\underset {(0.267)}{0.3438}$	$\underset {(0.0931)}{-0.2873^{***}}$	$\underset {(0.2505)}{0.3607}$	$\underset {(0.092)}{-0.3264^{***}}$
*Couple*	$\underset {(0.1613)}{-0.2004}$	$\underset {(0.0564)}{-0.0232}$	$\underset {(0.1533)}{-0.1646}$	$\underset {(0.0558)}{-0.0244}$
Male * Couple	$\underset {(0.2818)}{-0.0731}$	$\underset {(0.1059)}{0.5143^{***}}$	$\underset {(0.2649)}{-0.0802}$	$\underset {(0.1051)}{0.5179^{***}}$
*Number of children*	$\underset {(0.2837)}{-0.0196}$	$\underset {(0.0759)}{-0.6656^{***}}$	$\underset {(0.2812)}{-0.0488}$	$\underset {(0.0752)}{-0.6732^{***}}$
Male * Number of children	$\underset {(4048.322)}{-4.7589}$	$\underset {(0.147)}{0.4375^{***}}$	−	$\underset {(0.1454)}{0.4731^{***}}$
*No grade*	$\underset {(0.3068)}{0.7879^{**}}$	$\underset {(0.1034)}{-1.0041^{***}}$	$\underset {(0.2976)}{0.6897^{**}}$	$\underset {(0.1025)}{-1.0383^{***}}$
*College grade*	$\underset {(0.2439)}{0.5246^{**}}$	$\underset {(0.0793)}{-0.2972^{***}}$	$\underset {(0.2328)}{0.5132^{**}}$	$\underset {(0.0787)}{-0.3074^{***}}$
*High school grade*	$\underset {(0.2459)}{0.4477^{*}}$	$\underset {(0.0813)}{-0.285^{***}}$	$\underset {(0.236)}{0.423^{*}}$	$\underset {(0.0806)}{-0.2958^{***}}$
*Undergraduate studies*	$\underset {(0.238)}{0.545^{**}}$	$\underset {(0.094)}{0.1818^{*}}$	$\underset {(0.2272)}{0.5491^{**}}$	$\underset {(0.0931)}{0.1698^{*}}$
*Ref : Graduate studies*	-	-	-	-
Male * No grade	$\underset {(0.3735)}{-0.2022}$	$\underset {(0.1375)}{1.0893^{***}}$	$\underset {(0.3597)}{-0.1116}$	$\underset {(0.1361)}{1.1343^{***}}$
Male * College grade	$\underset {(0.2886)}{-0.2293}$	$\underset {(0.1006)}{0.4981^{***}}$	$\underset {(0.2726)}{-0.2672}$	$\underset {(0.0997)}{0.5252^{***}}$
Male * High school grade	$\underset {(0.3379)}{-0.4367}$	$\underset {(0.1143)}{0.1839}$	$\underset {(0.3205)}{-0.4298}$	$\underset {(0.1133)}{0.2008^{*}}$
Male * Undergraduate studies	$\underset {(0.3568)}{-0.4428}$	$\underset {(0.1349)}{-0.2258^{*}}$	$\underset {(0.3384)}{-0.5744^{*}}$	$\underset {(0.1316)}{-0.3314^{**}}$
Ref : Male * Graduate studies	-	-	-	-
*Medical density*	$\underset {(0.0012)}{0.0026^{**}}$	−	$\underset {(0.0011)}{0.001}$	−
*Unemployment rate*	−	$\underset {(0.0049)}{-0.005}$	−	$\underset {(0.0046)}{-0.0031}$
*Illness before professional life*	$\underset {(0.0062)}{0.0837^{***}}$	$\underset {(0.0046)}{-0.0006}$	$\underset {(0.0058)}{0.0823^{***}}$	$\underset {(0.0045)}{-0.0028}$
*Intercept*	$\underset {(1.5083)}{-3.8108^{**}}$	$\underset {(0.4429)}{0.1529}$	$\underset {(1.4725)}{-3.619^{**}}$	$\underset {(0.4377)}{0.2683}$
*λ*_11_	$\underset {(0.2498)}{1.8216^{***}}$	-		
*λ*_12_	$\underset {(0.0611)}{0.178^{***}}$	-		
*λ*_21_	-	$\underset {(0.1439)}{-0.9086^{***}}$		
*λ*_22_	-	$\underset {(0.0213)}{0.0627^{***}}$		
*Covariance matrix*	$\rho _{\epsilon } = \underset {(0.0639)}{-0.0748}$		-	

Standard errors are in parenthesis. ***: Significant at 1% level, **: Significant at 5% level. *: Significant at 10% level, +: Refers to the individual’s nationality

**Table 14:** Estimates of health and job status interactions. Part A: dynamic equations

	Bivariate estimations		Univariate estimations	
*Variables*	*h* : *rdeath*	*w* : *work*	*h* : *rdeath*	*w* : *work*
	(1)	(2)	(1’)	(2’)
*h*_−1_	$\underset {(0.0417)}{4.8659^{***}}$	$\underset {(0.0378)}{-0.4876^{***}}$	$\underset {(0.0632)}{4.6729^{***}}$	$\underset {(0.0422)}{-0.4136^{***}}$
*w*_−1_	$\underset {(0.0421)}{0.0402}$	$\underset {(0.0129)}{2.7146^{***}}$	$\underset {(0.0431)}{-0.0012}$	$\underset {(0.0137)}{2.716^{***}}$
*Age*	$\underset {(0.0106)}{0.0108}$	$\underset {(0.004)}{0.1068^{***}}$	$\underset {(0.011)}{0.0121}$	$\underset {(0.0042)}{0.1284^{***}}$
*Age square*	$\underset {(0.0001)}{0.00003}$	$\underset {(0.0001)}{-0.0017^{***}}$	$\underset {(0.0001)}{0.00003}$	$\underset {(0.0001)}{-0.0018^{***}}$
*Not French* ^+^	$\underset {(0.0465)}{-0.0829^{*}}$	$\underset {(0.0167)}{-0.3221^{***}}$	$\underset {(0.0479)}{-0.0806^{*}}$	$\underset {(0.0226)}{-0.2342^{***}}$
*Gender*(*male*)	$\underset {(0.0938)}{-0.0683}$	$\underset {(0.0573)}{0.3407^{***}}$	$\underset {(0.0963)}{-0.0673}$	$\underset {(0.0544)}{-0.003}$
*Couple*	$\underset {(0.0421)}{-0.0858^{**}}$	$\underset {(0.0173)}{-0.4812^{***}}$	$\underset {(0.0434)}{-0.1003^{**}}$	$\underset {(0.0185)}{-0.4144^{***}}$
Male * Couple	$\underset {(0.0657)}{0.0035}$	$\underset {(0.0309)}{0.7857^{***}}$	$\underset {(0.0682)}{0.0211}$	$\underset {(0.0323)}{0.6562^{***}}$
*Number of children*	$\underset {(0.0157)}{0.0119}$	$\underset {(0.0063)}{-0.156^{***}}$	$\underset {(0.0165)}{0.0056}$	$\underset {(0.0074)}{-0.1288^{***}}$
Male * Number of children	$\underset {(0.0229)}{-0.0099}$	$\underset {(0.0107)}{0.0254^{**}}$	$\underset {(0.0238)}{-0.01}$	$\underset {(0.0116)}{0.0234^{**}}$
*No grade*	$\underset {(0.0818)}{0.0269}$	$\underset {(0.0323)}{-0.8172^{***}}$	$\underset {(0.0846)}{0.0865}$	$\underset {(0.0427)}{-0.5912^{***}}$
*College grade*	$\underset {(0.0562)}{-0.0158}$	$\underset {(0.0255)}{-0.5311^{***}}$	$\underset {(0.0589)}{-0.0079}$	$\underset {(0.0309)}{-0.2774^{***}}$
*High school grade*	$\underset {(0.0656)}{0.027}$	$\underset {(0.0296)}{-0.3582^{***}}$	$\underset {(0.069)}{0.0264}$	$\underset {(0.036)}{-0.1875^{***}}$
*Undergraduate studies*	$\underset {(0.0758)}{0.018}$	$\underset {(0.0345)}{-0.1903^{***}}$	$\underset {(0.0781)}{0.0353}$	$\underset {(0.041)}{-0.1193^{***}}$
*Ref : Graduate studies*	-	-	-	-
Male * No grade	$\underset {(0.1209)}{0.1659}$	$\underset {(0.0654)}{-0.2214^{***}}$	$\underset {(0.1252)}{0.1253}$	$\underset {(0.0715)}{0.1816^{**}}$
Male * College grade	$\underset {(0.0891)}{0.0857}$	$\underset {(0.0557)}{-0.0569}$	$\underset {(0.0917)}{0.081}$	$\underset {(0.0545)}{0.1199^{**}}$
Male * High school grade	$\underset {(0.1037)}{0.1492}$	$\underset {(0.0647)}{-0.0928}$	$\underset {(0.108)}{0.1343}$	$\underset {(0.0653)}{0.0419}$
Male * Undergraduate studies	$\underset {(0.1292)}{-0.0181}$	$\underset {(0.0853)}{0.1564^{*}}$	$\underset {(0.1359)}{-0.0938}$	$\underset {(0.0778)}{0.0993}$
Ref : Male * Graduate studies	-	-	-	-
*Medical density*	$\underset {(0.0007)}{0.0011}$	−	$\underset {(0.0007)}{0.0009}$	−
*Unemployment rate*	−	$\underset {(0.0024)}{0.0428^{***}}$	−	$\underset {(0.0026)}{-0.0029}$
*Intercept*	$\underset {(0.1894)}{-3.4328^{***}}$	$\underset {(0.0699)}{-3.1154^{***}}$	$\underset {(0.2044)}{-3.5479^{***}}$	$\underset {(0.072)}{-2.1372^{***}}$
*Covariance matrix*	$\sigma _{1} = \underset {(0.0013)}{0.1656^{***}}$, $\sigma _{2} = \underset {(0.0161)}{1.7045^{***}}$		-	
	$\rho _{\eta } = \underset {(0.0062)}{-0.691^{***}}$, $\rho _{\zeta } = \underset {(0.0394)}{0.0214}$		-	

Standard errors are in parenthesis. ***: Significant at 1% level, **: Significant at 5% level. *: Significant at 10% level, +: Refers to the individual’s nationality

**Table 15:** Estimates of health and job status interactions. Part B: the initial conditions

	Bivariate estimations		Univariate estimations	
*Variables*	*h* : *rdeath*	*w* : *work*	*h* : *rdeath*	*w* : *work*
*Age*	$\underset {(0.1769)}{0.1328}$	$\underset {(0.0433)}{0.0808^{*}}$	$\underset {(0.1691)}{0.1306}$	$\underset {(0.0426)}{0.0848^{**}}$
*Age square*	$\underset {(0.0042)}{-0.003}$	$\underset {(0.0011)}{-0.0012}$	$\underset {(0.004)}{-0.0029}$	$\underset {(0.001)}{-0.0013}$
*Not French* ^+^	$\underset {(0.2722)}{-0.5346^{*}}$	$\underset {(0.0446)}{-0.4653^{***}}$	$\underset {(0.2552)}{-0.4894^{*}}$	$\underset {(0.0442)}{-0.4486^{***}}$
*Gender*(*male*)	$\underset {(0.2569)}{-0.008}$	$\underset {(0.0933)}{-0.3^{***}}$	$\underset {(0.2444)}{-0.037}$	$\underset {(0.092)}{-0.3264^{***}}$
*Couple*	$\underset {(0.156)}{-0.0629}$	$\underset {(0.0565)}{-0.0209}$	$\underset {(0.1472)}{-0.01}$	$\underset {(0.0558)}{-0.0244}$
Male * Couple	$\underset {(0.2616)}{0.2684}$	$\underset {(0.106)}{0.5006^{***}}$	$\underset {(0.2493)}{0.1961}$	$\underset {(0.1051)}{0.5179^{***}}$
*Number of children*	$\underset {(0.2928)}{-0.0773}$	$\underset {(0.0761)}{-0.6625^{***}}$	$\underset {(0.2743)}{-0.0694}$	$\underset {(0.0752)}{-0.6732^{***}}$
Male * Number of children	$\underset {(171.3052)}{-3.4294}$	$\underset {(0.1475)}{0.442^{***}}$	$\underset {(0)}{0^{***}}$	$\underset {(0.1454)}{0.4731^{***}}$
*No grade*	$\underset {(0.2863)}{0.4708}$	$\underset {(0.1035)}{-0.995^{***}}$	$\underset {(0.278)}{0.446}$	$\underset {(0.1025)}{-1.0383^{***}}$
*College grade*	$\underset {(0.2251)}{0.0628}$	$\underset {(0.0795)}{-0.2944^{***}}$	$\underset {(0.2154)}{0.0935}$	$\underset {(0.0787)}{-0.3074^{***}}$
*High school grade*	$\underset {(0.2227)}{0.0513}$	$\underset {(0.0814)}{-0.2885^{***}}$	$\underset {(0.2138)}{0.0653}$	$\underset {(0.0806)}{-0.2958^{***}}$
*Undergraduate studies*	$\underset {(0.2422)}{-0.083}$	$\underset {(0.0942)}{0.1787^{*}}$	$\underset {(0.2305)}{-0.0627}$	$\underset {(0.0931)}{0.1698^{*}}$
*Ref : Graduate studies*	-	-	-	-
Male * No grade	$\underset {(0.4161)}{-0.3545}$	$\underset {(0.1378)}{1.0946^{***}}$	$\underset {(0.4134)}{-0.3361}$	$\underset {(0.1361)}{1.1343^{***}}$
Male * College grade	$\underset {(0.2849)}{-0.0068}$	$\underset {(0.1009)}{0.4915^{***}}$	$\underset {(0.2723)}{-0.0121}$	$\underset {(0.0997)}{0.5252^{***}}$
Male * High school grade	$\underset {(0.3535)}{-0.3165}$	$\underset {(0.1145)}{0.1914^{*}}$	$\underset {(0.3406)}{-0.304}$	$\underset {(0.1133)}{0.2008^{*}}$
Male * Undergraduate studies	$\underset {(0.3775)}{-0.0238}$	$\underset {(0.1332)}{-0.3359^{**}}$	$\underset {(0.3606)}{-0.0475}$	$\underset {(0.1316)}{-0.3314^{**}}$
Ref : Male * Graduate studies	-	-	-	-
*Medical density*	$\underset {(0.0013)}{0.0028^{**}}$	−	$\underset {(0.0012)}{0.0016}$	−
*Unemployment rate*	−	$\underset {(0.005)}{-0.0064}$	−	$\underset {(0.0046)}{-0.0031}$
*Illness before professional life*	$\underset {(0.0067)}{0.0754^{***}}$	$\underset {(0.0046)}{-0.001}$	$\underset {(0.0063)}{0.0725^{***}}$	$\underset {(0.0045)}{-0.0028}$
*Intercept*	$\underset {(1.8164)}{-4.7064^{**}}$	$\underset {(0.4432)}{0.134}$	$\underset {(1.7456)}{-4.3378^{**}}$	$\underset {(0.4377)}{0.2683}$
*λ*_11_	$\underset {(0.2998)}{1.6027^{***}}$	-		
*λ*_12_	$\underset {(0.0635)}{0.2177^{***}}$	-		
*λ*_21_	-	$\underset {(0.1524)}{-1.1728^{***}}$		
*λ*_22_	-	$\underset {(0.0223)}{0.0398^{***}}$		
*Covariance matrix*	$\rho _{\epsilon } = \underset {(0.0719)}{-0.0692}$		-	

Standard errors are in parenthesis. ***: Significant at 1% level, **: Significant at 5% level. *: Significant at 10% level, +: Refers to the individual’s nationality

## Abbreviations

By order of appearance. DARES: Direction de l’Animation de la Recherche, des Etudes et des Statistiques
DREES: Direction de la Recherche, des Etudes, de l’Evaluation et des Statistiques
ICD: International classification of diseases
INSEE: Institut National de Statistique et d’Etudes Economiques
IRDES: Institut de Recherche et de Documentation en Economie de la Santé
SIP: Santé et Itinéraire Professionnel

## Footnotes

1 Excluding the initial lagged period.

2 International Statistical Classification of Diseases and Related Health Problems - 10th Revision (ICD-10)

3 Among all these variables, only three (age, number of children, and marital status) vary over time.

4 [54] provide more details about how to make this choice.

5 In order to take the remark of one anonymous referee on the effect of health shocks (changes in disability index or in death risks) on job status, we have created two dummies variables: *D*1=1 if disability index changes from 0 to 1 between *t*−1 and *t*, *D*1=0 otherwise and *D*2=1 if death risks changes from 0 to 1 between *t*−1 and *t*, *D*2=0 otherwise. Univariate estimations (available on request) shows no effects of these two variables on job status.

6 Such as mental health [55, 56]
